# Evaluation of risk prediction scores for adults hospitalized with COVID-19 in a highly-vaccinated population, Aotearoa New Zealand 2022

**DOI:** 10.1016/j.ijregi.2024.100424

**Published:** 2024-08-13

**Authors:** Michael James Maze, Jonathan Williman, Rebekah Anstey, Emma Best, Hasan Bhally, Aliya Bryce, Catherina L. Chang, Kevin Chen, Jack Dummer, Michael Epton, William R. Good, Jennifer Goodson, Corina Grey, Kate Grimwade, Robert J. Hancox, Redzuan Zarool Hassan, Thomas Hills, Sandra Hotu, Colin McArthur, Susan Morpeth, David R. Murdoch, Fiona Elizabeth Pease, Romana Pylypchuk, Nigel Raymond, Stephen Ritchie, Deborah Ryan, Vanessa Selak, Malina Storer, Tony Walls, Rachel Webb, Conroy Wong, Karen Wright

**Affiliations:** a Department of Medicine, University of Otago, Christchurch, New Zealand; b Department of Population Health, University of Otago, Christchurch, New Zealand; c Respiratory Department, Te Whatu Ora Waikato, New Zealand; d Department of Paediatrics, University of Auckland, New Zealand; e Infectious Diseases Department, Te Whatu Ora Waitematā, New Zealand; f Infectious Diseases Department, Te Whatu Ora Hauora a Toi Bay of Plenty, New Zealand; g Department of Medicine, University of Otago, New Zealand; h Respiratory Department, Te Whatu Ora Waitaha Canterbury, New Zealand; i Respiratory Department, Te Whatu Ora Counties Manukau, New Zealand; j Department of General Practice and Primary Healthcare, University of Auckland, New Zealand; k Department of Preventive and Social Medicine, University of Otago, New Zealand; l Department of Infectious Diseases, Te Whatu Ora Auckland, New Zealand; m Medical Research Institute of New Zealand, Wellington, New Zealand; n Respiratory Medicine Department, Te Whatu Ora Auckland, New Zealand; o Department of Critical Care Medicine, Te Whatu Ora Auckland, New Zealand; p Department of Infectious Diseases, Te Whatu Ora Counties Manukau, New Zealand; q Department of Pathology and Biomedical Science, University of Otago, Christchurch, New Zealand; r Department of Epidemiology and Biostatistics, University of Auckland, New Zealand; s Infection Service, Te Whatu Ora Capital, Coast and Hutt Valley, New Zealand; t Pacific Perspectives, Wellington, New Zealand; u Department of Paediatrics, University of Otago, Christchurch, New Zealand; v Faculty of Medical and Health Sciences, University of Auckland, New Zealand; w Te Kupenga Hauora Māori, University of Auckland, New Zealand

**Keywords:** COVID-19, SARS-CoV-2, Minority health, Māori health, Pacific health

## Abstract

**Objectives:**

COVID-19 severity prediction scores need further validation due to evolving COVID-19 illness. We evaluated existing COVID-19 risk prediction scores in Aotearoa New Zealand, including for Māori and Pacific peoples who have been inequitably affected by COVID-19.

**Methods:**

We conducted a multicenter retrospective cohort study in adults hospitalized with COVID-19 from January to May 2022, including all Māori and Pacific patients, and every second non-Māori, non-Pacific (NMNP) patient to achieve equal analytic power by ethnic grouping. We assessed the accuracy of existing severity scores (4C Mortality, CURB-65, PRIEST, and VACO) to predict death in the hospital or within 28 days.

**Results:**

Of 2319 patients, 582 (25.1%) identified as Māori, 914 (39.4%) as Pacific, and 862 (37.2%) as NMNP. There were 146 (6.3%, 95% confidence interval 5.4-7.4%) deaths, with a predicted probability of death higher than observed mortality for VACO (10.4%), modified PRIEST (15.1%) and 4C mortality (15.5%) scores, but lower for CURB-65 (4.5%). C-statistics (95% CI) of severity scores were: 4C mortality: Māori 0.82 (0.75, 0.88), Pacific 0.87 (0.83, 0.90), NMNP 0.90 (0.86, 0.93); CURB-65: Māori 0.83 (0.69, 0.92), Pacific 0.87 (0.82, 0.91), NMNP 0.86 (0.80, 0.91); modified PRIEST: Māori 0.85 (0.79, 0.90), Pacific 0.81 (0.76, 0.86), NMNP 0.83 (0.78, 0.87); and VACO: Māori 0.79 (0.75, 0.83), Pacific 0.71 (0.58, 0.82), NMNP 0.78 (0.73, 0.83).

**Conclusions:**

Following re-calibration, existing risk prediction scores accurately predicted mortality.

## Highlights

• In our cohort of patients hospitalized due to COVID-19, 6.3% died.
• CURB-65, 4C Mortality, and PRIEST scores all accurately predicted mortality.
• Risk prediction scores were accurate for Māori and Pacific peoples.

## Introduction

Clinicians need to assess the severity of illness in patients with COVID-19 when making decisions regarding triage, hospital admission, inpatient ward distribution, and therapeutics. Several risk prediction scores have been developed or repurposed to predict the likelihood of severe COVID-19 based on vital signs, demographic factors, or co-morbidities [[1], [2], [3], [4]]. Risk prediction scores may be useful for identifying patients at very low risk of severe illness who might be suitable for community management or early discharge, but evaluations are needed. In addition, there is uncertainty about the utility of existing risk prediction scores in the Aotearoa New Zealand context where the effects of colonization, racism, and privilege influence the distribution of disease and outcomes [5], and where COVID-19 illness has evolved considerably since the initial descriptions with reduced disease severity due to exposure, vaccination, and evolving SARS-CoV-2 variants.

Pervasive and persistent health inequities in Aotearoa New Zealand are well described for Māori and Pacific peoples when compared to non-Māori, non-Pacific (NMNP) peoples [6,7]. Over the duration of the COVID-19 pandemic, unfair and unjust disparities in vaccination status, availability of accurate vaccination coverage information, public health response, infection rates, hospitalization, and mortality have been reported [8,9]. Determinants of ethnic inequities are complex but are created by three main pathways: (i) differential access to the determinants of health, (ii) differential access to health care, and (iii) differences in quality of care [10,11].

Assessment of disease severity at hospital admission has the potential to reduce or increase inequity of outcomes by influencing clinical decision-making. In this context, it is essential that any COVID-19 severity prediction scores accurately reflect the risk for Māori and Pacific peoples, recognizing that Māori are the indigenous peoples of Aotearoa New Zealand, and that both populations have been disproportionately impacted by higher infection rates and adverse COVID-19 outcomes.

Our study aimed to validate prognostic severity prediction scores for adults hospitalized with COVID-19 in a way that supports equitable outcomes, particularly for Māori and Pacific peoples.

## Methods

### Study design

We performed a retrospective cohort study of a sample of patients hospitalized with COVID-19 at 11 hospitals across Aotearoa New Zealand. The study is reported according to transparent reporting of a multivariable prediction model for individual prognosis or diagnosis (TRIPOD) [12], and the consolidated criteria for strengthening reporting of health research involving indigenous peoples (CONSIDER) guidelines [13].

### Study governance

The study has centered Te Tiriti o Waitangi (the Māori version of the Treaty of Waitangi, the founding document establishing the relationship between Māori and the British Crown) in its research design and implementation. Specific measures included Māori health leadership and an Equity Expert Reference Group, containing Pacific health and Māori health expertise, to inform study design, data analysis, and the final report. The study gained approval from The Northern B Regional Health and Disability Ethics Committee (20NTB72).

### Study population

Our study included patients admitted from January 1 to May 1, 2022. Eligible patients were identified as being adults (aged ≥16 years) diagnosed with COVID-19 within the 14 days prior to or 2 days after the date of acute hospital admission. We included only patients whose hospitalization was attributable to SARS-CoV-2 infection (Supplementary Methods).

### Sampling strategy

The required sample size was estimated *a priori* in March 2022. Prior estimates were that case fatality of hospitalized patients was approximately 11·5% (95% confidence interval [CI] 7.7-16.9%) [14]. Therefore, we sampled ∼2500 patients, of whom we estimated 250 would die. We estimated this would allow concordance statistic (C-statistic) for severity prediction scores to be estimated with a 95% CI of ± 4% [15]. The goal of sampling was that model validation would have equal explanatory power for Māori, Pacific peoples, and NMNP peoples. To achieve this, we retrieved clinical notes at each study site for confirmation of eligibility and collection of study data from all patients of Māori and/or Pacific ethnicity, and every second patient of NMNP ethnicity (ordered by admission date and time). In this way, we oversampled Māori and Pacific patients, and therefore incidence and case fatality cannot be estimated from this study cohort.

### Choice of risk prediction scores

We identified risk prediction scores that had demonstrated sufficient accuracy on previously published evaluations for COVID-19 and contained variables that indicated they could be implemented within the Aotearoa New Zealand health system (Table 1). These included 4C mortality [1], CURB-65 [16], VACO [4], and PRIEST scores [2]. As performance status was not routinely recorded within hospital records, the PRIEST score was modified by the removal of this criteria.

**Table 1:** Relevant mortality risk prediction tools for use in patients with COVID-19.

Prediction tool	Original outcome	Predictors included in models
4C Mortality	In hospital mortality	Age, sex, number of co-morbidities, respiratory rate, oxygen saturation on room air, Glasgow coma scale, blood urea, C-reactive protein
CURB-65	30-day mortality	Confusion, blood urea, respiratory rate, blood pressure, age
VACO	30-day mortality	Age, sex, Charlson co-morbidity index, myocardial infarction, or peripheral vascular disease
PRIEST	30-day mortality or major organ support	Age, sex, respiratory rate, oxygen saturation, heart rate, systolic blood pressure, temperature, alertness, inspired oxygen, and performance status.

### Data collection and outcomes

The study team used a standardized data collection form to extract information from clinical records about patient demographics, symptoms, and signs on presentation to the hospital, existing co-morbidities, usual medications, specific COVID-19 treatments, complications of COVID-19 and outcomes in hospital and at 28 days. Data collected using the study case report form were supplemented by Ministry of Health routine datasets to improve data quality for key variables. Specifically, we linked data (National Hospital Identifier with the National Hospital Index dataset, the Mortality Collection dataset, and the National Minimum Dataset) to supplement information regarding ethnicity and death. Classification of ethnicity was by 2018 New Zealand Census categories, defined as the ethnic group(s) to which people identify [17]. Ethnicity was manually obtained from electronic and paper-based hospital records. Fields for up to six ethnic groups were included and study staff were directed to enter all ethnic groups recorded in hospital records. Level 1 ethnicity groupings (the broadest level) were used to create the ethnicity variables for analyses of Māori, Pacific peoples, and NMNP (Asian, MELAA [Middle Eastern/Latin American/African], New Zealand European, and Other) [17]. The Pacific peoples’ ethnic group is an umbrella term that includes people descended from the indigenous peoples of Pacific Island countries. The five largest Pacific groups in Aotearoa New Zealand include Samoans, Tongans, Cook Islands Māori, Niueans, and Fijians. The unit of analysis for the study was an episode of care and only the first hospitalization due to COVID-19 during the study period was included in our analysis. Our primary outcome was death either while an inpatient or within 28 days of admission.

Data extraction and data entry were performed by study staff who either were clinicians (nursing or medical) or final year (6^th^ year) medical students. All research staff completed training to standardize attributing causes of admission and completed the International Conference on Harmonization Good Clinical Practice certification. Data integrity was assured by double data entry of the first 2-3 cases for each study staff inputting data, inbuilt quality rules within the database, and centralized data cleaning checks to ensure values were plausible and consistent. Where necessary, values were rechecked in the clinical record.

### Statistical analysis

Data was stored in a REDCap database, and analyzed in R version 4.2.2 [18]. Predicted probabilities of adverse outcomes were calculated for each score, following the directions of the score creator as closely as possible. Where risk prediction scores could be calculated for fewer than 90% of cases, missing data was multiply imputed via chain equations using the Multivariate Imputation by Chained Equations (MICE) package (version 3.16.0) to create 10 datasets each with a run-in of 20 iterations [19]. In addition, complete case analyses were performed as sensitivity analyses. Logistic regression models were used to impute binary variables and predictive mean matching for continuous variables. Variables included in the imputation datasets included only the outcome of interest and variables needed for the calculation of the risk prediction scores. Highly skewed continuous variables were considered for transformation prior to imputation. For each of the risk prediction scores, individuals’ predicted mortality risks were calculated across all 10 imputed datasets and then averaged prior to deriving validation statistics.

Model performance was assessed for each combination of risk prediction tool and observed mortality through calibration curves (CalibrationCurves package 1.0.0), including flexible and logistic calibration curves to allow comparison against the ideal fit. We assessed severity prediction scores using overall performance measures (explained variation, Brier's score), calibration (observed/expected ratio, overall average miscalibration, calibration slope, and intercept), discrimination (area under the receiver-operator curve also known as c-statistic), and clinical utility (sensitivity, specificity, positive predictive value [PPV] and negative predictive values [NPV]). Validation was stratified by total response ethnicity (Māori, Pacific, NMNP), and heterogeneity of model performance measures by subgroup was assessed. We evaluated the potential utility of scores for ‘ruling out’ poor outcomes by establishing cut-points for models producing scores (4C mortality, CURB-65, and modified PRIEST) that indicate a <1% probability of death.

Data-driven updating of the models was performed through re-calibration of the intercept and coefficient, re-estimation of model coefficients, and the addition of new predictors as described elsewhere [20]. Re-estimation of model coefficients was undertaken by fitting the items of each risk prediction score in logistic regression models for each of the 10 imputed datasets, using Rubin's rules to pool estimates. The performance of original, re-calibrated, and re-estimated risk prediction scores were compared using likelihood ratio tests. To limit the risk of model overfitting, the addition of new predictors was approached cautiously. Vaccination status (as a binary variable indicating ≥2 doses of a COVID-19 vaccine) and presence of co-morbidities were chosen *a priori* and assessed for inclusion. Vaccination status is potentially associated with death but had not been tested during the original score development; the number of co-morbidities was included in some but not all scores.

## Results

We identified 2319 admissions attributable to COVID-19. Of the patients included, 582 (25.1%) identified as Māori, and 914 (39.4%) identified as Pacific peoples. We classified 862 (37.2%) as NMNP, including participants who identified as New Zealand European 689 (29.7%), Asian 140 (6.0%), MELAA 47 (2.0%) and other 9 (0.4%). Among included hospitalizations, 2054 (88.6%) patients were recorded as identifying as a single ethnicity, however, 145 (24.9%) of 582 Māori and 91 (10.0%) Pacific patients were recorded as identifying with more than one Level 1 ethnicity. Clinical and demographic characteristics by ethnicity are presented in Supplementary Table S1. Overall, 1828 (78.8%) patients had received ≥2 doses of COVID-19 vaccine, including 427 (73.4%) Māori and 701 (76.7%) Pacific patients.

The median (interquartile range) length of hospital stay was 2 days (1-5 days), with 79 (3.4%) admitted to an intensive care unit and 146 (6.3%) dying during the hospital admission or within 28 days. Table 2 indicates the clinical characteristics of included patients by outcome. Prior to admission, 71 (3.1%) of patients had received COVID-19-specific antiviral medication. During hospital admission 1261 (54.4%) received either prophylactic or therapeutic anticoagulation, 904 (38.4%) received oral or intravenous corticosteroids, 294 (12.7%) received COVID-19 specific antiviral medications, 163 (7.0%) received immune-modulators (tocilizumab or baricitinib).

**Table 2:** Clinical characteristics of adults admitted to hospital due to COVID-19 by outcome, Aotearoa New Zealand, 2022.

Characteristic	Observed	Survivors (N = 2173)		Decedents (N = 146)	
	n (%)	n	(%)	n	(%)
Age at admission, median (interquartile range)	2319 (100%)	56	(36,72)	79	(66.0,85.0)
Female sex	2319 (100%)	1309	(60.2%)	62	42.5%
Male sex	2319 (100%)	864	(39.8%)	84	57.5%
Symptoms onset until admission (days), median (interquartile range)	2308 (100%)	3	(1,6)	3	(1.0,7.0)
Pregnant	2319 (100%)	197	(7.3%)	1	0.7%
Number of previous COVID vaccinations	2319 (100%)				
- None		387	(14.3%)	28	19.2%
- One vaccination		72	(2.7%)	4	2.7%
- Two vaccinations		846	(31.2%)	50	34.2%
- ≥Three vaccinations		868	(32.0%)	64	43.8%
Smoking status	2014 (87%)				
- Never smoked		942	(34.7%)	46	31.5%
- Ex-smoker		657	(24.2%)	55	37.7%
- Current smoker		307	(11.3%)	7	4.8%
Co-morbidities	2309 (100%)				
- 0		621	(22.9%)	5	3.4%
- 1		624	(23.0%)	40	27.4%
- 2		478	(17.6%)	36	24.7%
- 3		291	(10.7%)	36	24.7%
- 4		159	(5.9%)	29	19.9%
AVPU	1991 (86%)				
- Alert		1823	(67.2%)	98	67.1%
- Verbal		35	(1.3%)	13	8.9%
- Pain		7	(0.3%)	6	4.1%
- Unresponsive		8	(0.3%)	1	0.7%
Glasgow coma scale < 15	1194 (51%)	158	(5.8%)	53	36.3%
Temperature	2265 (98%)				
- 36.1-38.0		1440	(53.1%)	95	65.1%
- <36.0 or >38.0		681	(25.1%)	49	33.6%
Heart rate	2295 (99%)				
- 51-90		1308	(48.2%)	81	55.5%
- 41-50 or 91-110		22	(0.8%)	1	0.7%
- 111-130		211	(7.8%)	13	8.9%
- <41 or >130		60	(2.2%)	7	4.8%
Systolic blood pressure	2232 (96%)				
- 91-100		104	(3.8%)	17	11.6%
- 111-219		1686	(62.1%)	94	64.4%
- 101-110		246	(9.1%)	16	11.0%
- <91 or >219		55	(2.0%)	14	9.6%
Respiratory rate	2290 (99%)				
- 9-11		3	(0.1%)	1	0.7%
- 12-20		1289	(47.5%)	57	39.0%
- 21-24		486	(17.9%)	45	30.8%
- <9 or >24		367	(13.5%)	42	28.8%
Oxygen saturation <92%	2071 (89%)	147	(5.4%)	25	17.1%
Blood urea	1764 (76%)				
- <7		1084	(40.0%)	31	21.2%
- ≥7		552	(20.4%)	97	66.4%
C-reactive protein	2004 (86%)				
- <50		1417	(52.2%)	58	39.7%
- 51-99		227	(8.4%)	36	24.7%
- >100		224	(8.3%)	42	28.8%

**Figure 1:**
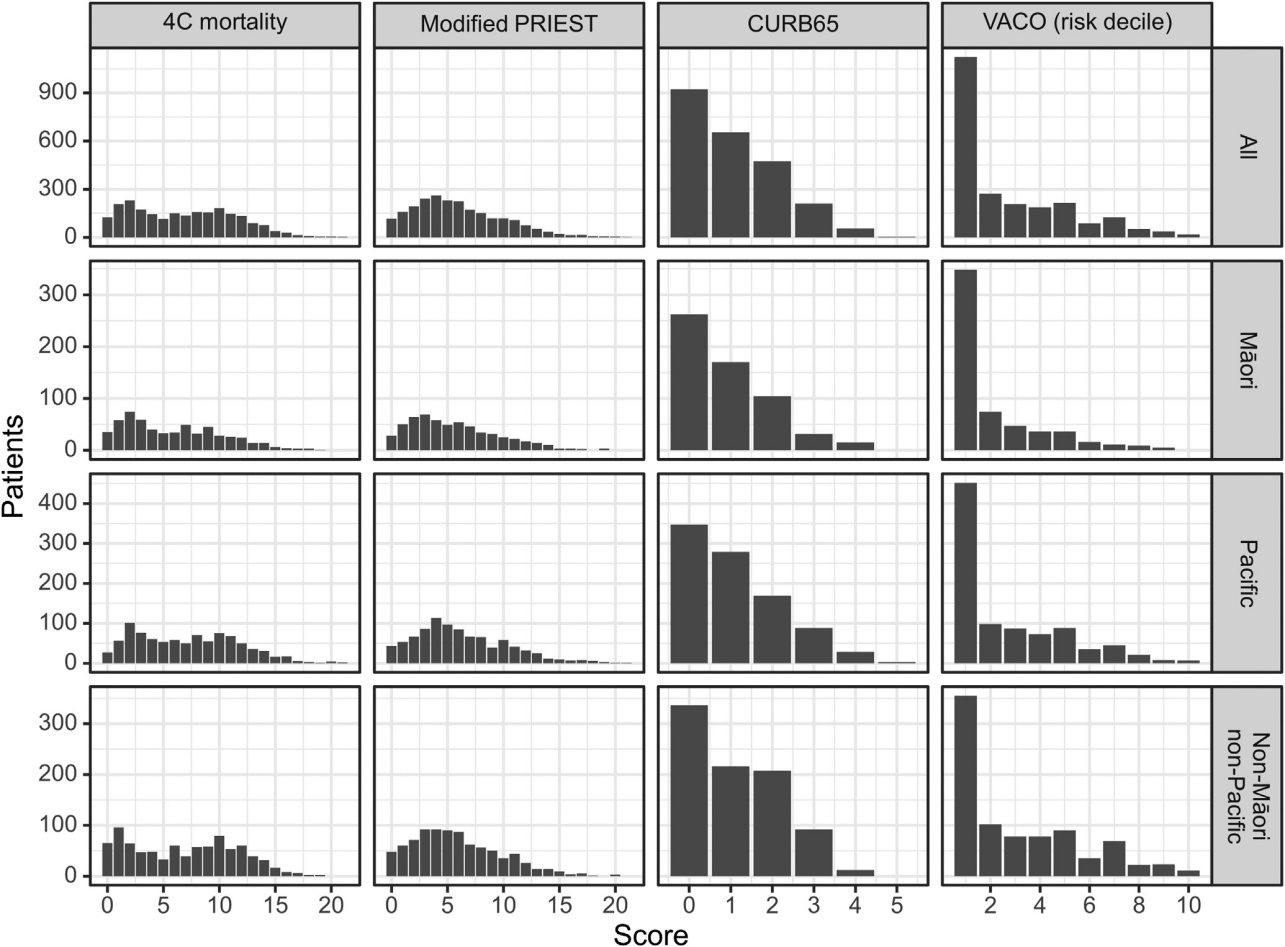
Distribution of COVID-19 risk prediction scores in adults (aged ≥16 years) admitted to hospital due to COVID-19 by Māori, Pacific, and non-Māori non-Pacific, Aotearoa New Zealand, 2022.

Figure 1 indicates the distribution of scores for each of the risk prediction tools. The mean predicted probability of death was notably higher than the observed mortality rate of 6.3% (95% CI 5.4-7.4%) for the VACO (10.4 %), modified PRIEST (15.1%) and 4C mortality (15.5%) scores, but lower for the CURB-65 (4.5%). Full calibration indices are included in Supplementary Table S2. Re-calibration of the intercept and slope improved the correlation between predicted mortality risk and observed mortality (Supplementary Table S2). Odds ratios were estimated for each predictor in the model (Supplementary Tables S3-S6). The addition of vaccination status and co-morbidity were considered due to the clinical plausibility of the effect. Vaccination status did not improve model fit for the 4C mortality (*P* = 0.76), modified PRIEST (*P* = 0.80), CURB-65 (*P* = 0.97), or VACO (*P* = 0.66) models. The addition of ≥1 co-morbidity was associated with increased risk of death odds ratio = 5.10 (95% CI 2.05-12.7, *P* <0.001) when included in the modified PRIEST score and when added to the CURB-65 score: odds ratio = 3.30 (95% CI 1.30-8.36, *P* = 0.012).

**Figure 2:**
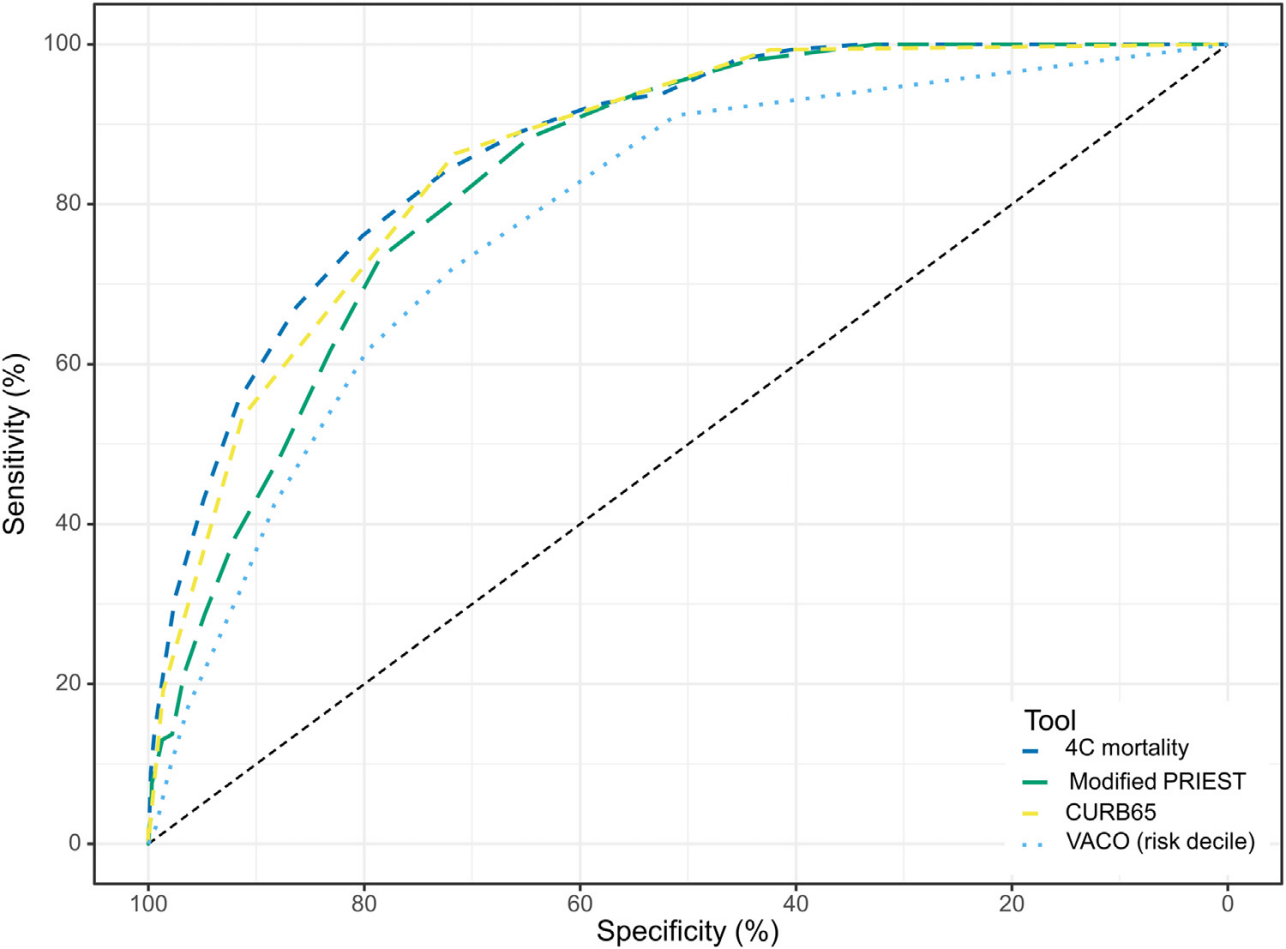
Receiver-operator curve of selected COVID-19 risk prediction scores in adults (aged ≥16 years) admitted to hospital due to COVID-19, 2022.

The receiver-operator curve (Figure 2) and C-statistics (Table 3) indicate 4C Mortality (0.87, 95% CI 0.84-0.90), CURB-65 (0.86, 95% CI 0.82-0.89) and modified PRIEST (0.83, 95% CI 0.80-0.85) each had a high degree of accuracy. Although C-statistic point estimates of 4C mortality and CURB-65 were lower for Māori, CIs for each overlapped Pacific and NMNP groups. Model estimates using complete case analysis are shown in Supplementary Table S7, demonstrating overlapping confidence intervals with the main analysis for each risk prediction score.

**Table 3:** Estimated concordance-statistic (95% confidence intervals) of selected updated severity prediction scores in adults (aged ≥16 years) admitted to hospital due to COVID-19 by Māori, Pacific, and non-Māori non-Pacific, Aotearoa New Zealand, 2022.

Model	All	Māori	Pacific	Non-Māori, non-Pacific
4C mortality	0·87 (0·84, 0·89)	0·82 (0·75, 0·88)	0·87 (0·83, 0·90)	0·90 (0·86, 0·93)
CURB-65	0·86 (0·82, 0·89)	0·83 (0·69, 0·92)	0·87 (0·82, 0·91)	0·86 (0·80, 0·91)
modified PRIEST	0·83 (0·80, 0·85)	0·85 (0·79, 0·90)	0·81 (0·76, 0·86)	0·83 (0·78, 0·87)
VACO	0·79 (0·75, 0·83)	0·71 (0·58, 0·82)	0·78 (0·73, 0·83)	0·83 (0·77, 0·87)

PRIEST score modified by removal of performance status.

In models that produce a score, cut-points could be chosen to identify patients with a very low probability of death. For example, patients with a 4C score <8 had a sensitivity = 92.5%, specificity = 58.5%, PPV = 13.0%, NPV = 99.1%, 0.9% probability of death and included 55.3% of the study cohort; a modified PRIEST score <6 had a sensitivity = 93.8%, specificity = 54.8%, PPV = 12.2%, NPV = 99.2%, 0.8% probability of death and included 51.7% of the study cohort, and a CURB-65 <1 had an NPV = 99.8%, 0·1% probability of death (Supplementary Tables S8-S10) and included 39.2% of the study population.

## Discussion

Prior to January 2022, Aotearoa New Zealand had recorded only 2.5 cases of COVID-19 per 1000 people, and 0.01 COVID-19-related deaths per 1000 due to closed borders and a strategy of eliminating infections [21]. Māori and Pacific peoples had higher hospitalization and twice the mortality rate of European and other ethnic groups [9]. In January 2022, the B1.1.529 (Omicron) variant spread rapidly through the community. At this time, 90% of people aged >12 years had received two doses of Pfizer/BioNTech BNT162b2 vaccine and 27% of the population (35% of adults) had received a third dose. However, vaccine coverage was inequitable with lower proportions of Māori and Pacific peoples receiving two doses [21]. Therefore, our study evaluates risk prediction scores in a highly-vaccinated but infection-naïve population hospitalized due to COVID-19 during a time period when the SARS-CoV-2 Omicron variant was prevalent. In this context, the clinical picture of COVID-19 has changed considerably from the original descriptions. We found that, following re-calibration, several risk prediction scores performed with a high degree of accuracy and remained suitable for risk prediction across each ethnic group.

Patients in our study experienced a substantially different illness than that described in early reports of COVID-19. We found patients presented to the hospital early in their illness, with a median 3 days of symptoms, substantially shorter than the 7-day median duration of symptoms in 2020 [22]. While COVID-19 continued to cause respiratory complications, only a quarter of patients had evidence of pneumonitis on chest radiography or required oxygen, and complications such as delirium and acute kidney injury were more commonly seen. Our cohort of patients had a high frequency of co-morbidities, consistent with international data that have shown a shift toward a predominance of presentations by older, more co-morbid patients [23].

We found that the inpatient fatality rate was, at 3·5%, substantially lower than during 2020 in Aotearoa New Zealand (12%) [22], and contemporaneous international comparisons. For example, in the United States, from January to June 2022, 11·8% of those hospitalized with COVID-19 died [23]. The criteria and threshold for hospital admission may be an important factor in the observed differences. There are many possible additional reasons for the observed differences in mortality with United States data including SARS-CoV-2 vaccination against COVID-19 [24], and comparatively lower hospital occupancy rates [25]. Similarly, the predominance of the Omicron SARS-CoV-2 variant [26], COVID-19 vaccination, and the availability of evidence-based treatments are likely to have contributed to the decrease in inpatient fatality in Aotearoa New Zealand compared to earlier in the pandemic. However, we did note that guideline-based treatments were not universally applied, with just over half of all patients receiving anticoagulant medication. We did not identify a difference in age-standardized case fatality rates for each ethnic grouping in our cohort. We highlight that this reflects only the risk among those hospitalized due to COVID-19 within our study sites, whereas existing data have identified a greater risk of hospitalization for Māori and Pacific peoples, and greater overall case fatality among those infected [8,27]. Therefore, work to reduce inequities in access to health care, quality of care, co-morbidities, and socio-economic determinants remain critical.

The evaluated severity prediction scores showed a high level of discrimination in predicting mortality. With large differences in observed mortality compared to previous studies, re-calibration was needed to accurately reflect risk, but the discriminative ability of predictive scores was similar. We considered the addition of vaccination status to prediction scores, but it did not increase performance. The 4C mortality score was validated internationally early in the pandemic as an accurate risk prediction tool and our findings further validate its use in a highly-vaccinated population infected with the Omicron variant [1,28]. Notably, the c-statistic in our study was higher than previously estimated. Although the c-statistic point estimate was lower for Māori, confidence intervals overlap with those of other evaluated ethnic groups. The CURB-65 score, commonly used for prediction of mortality risk for patients admitted with community-acquired pneumonia, performed with similar discriminatory ability to specifically developed scores for COVID-19, showing a high degree of accuracy in predicting which patients would go on to die. The c-statistic was substantially higher than seen in earlier validation studies (e.g., 0.72 [95% CI 0.71-0.73]) [1]. Previous critiques of the CURB-65 score in patients hospitalized with COVID-19 were that it substantially underestimated the risk of death and that a greater focus on markers of respiratory collapse was needed [3]. The evolution of the clinical illness from severe pneumonitis seen early in the pandemic to a disease with similar complications and mortality to community-acquired pneumonia [16], may be behind the improved accuracy seen in our study. The PRIEST score, modified to exclude functional status, was as accurate as the 4C mortality score in our population and was the best-performing score for Māori. The C-statistic in our study was higher (0.83, 95% CI 0.80-0.86) than that in the original derivation and validation study in the United Kingdom where the primary outcome was death or organ support and similar to a subsequent smaller evaluation in the United States (0.86, 95% CI 0.81-0.91) [2,29].

The 4C mortality, CURB-65, and modified PRIEST scores may each be suitable for implementation within hospitals in Aotearoa New Zealand to predict the risk of death in patients admitted with COVID-19. The included parameters are intuitive for clinicians, readily collected, and calculation is simply performed. Each has a high c-statistic and correlation between predicted and observed mortality, and a threshold score below which patients have a very low risk of death.

Our study has a number of limitations that influence interpretation. First, the relatively low case fatality resulted in the inclusion of relatively few deaths, particularly among Māori. Consequently, there is reduced confidence in estimates of risk prediction score accuracy for Māori. Second, we did not ascertain the infecting SARS-CoV-2 variant for patients. During the study period, the Omicron variant predominated but some patients are likely to have been infected with the Delta variant [30]. Third, we employed a retrospective study design and data was incomplete for some variables within the risk prediction scores, notably level of consciousness and blood urea concentrations. We mitigated this through the use of multiple imputations but the potential for bias still exists. Finally, our study population included only patients admitted to a hospital with a preponderance of hospitals in larger population centers. As such may be less applicable to patients presenting to rural hospitals or in primary care.

In conclusion, among a highly-vaccinated population likely infected with Omicron variant SARS-CoV-2, patients hospitalized with COVID-19 experienced a multisystem disease, with pneumonitis or respiratory failure only seen in a minority. Despite a substantially lower case fatality than previously reported, existing risk prediction scores, once re-calibrated, performed with a high degree of accuracy. Further, they show similarly high accuracy among Māori and Pacific peoples as they do among NMNP peoples. Objective assessment of risk among Māori and Pacific peoples can support accurate decision-making around discharge and therapeutics and has the potential to reduce inequity. The evaluated risk prediction scores contain clinical information that is routinely collected by clinicians and are relatively simple to calculate. We consider them appropriate for implementation in clinical practice to identify patients at very low risk of death when considering disposition following assessment in the hospital. Implementation requires input from multidisciplinary clinicians regarding integration into clinical practice and further evaluation of any score implemented is needed to confirm the score performs well for all and does not act to increase or maintain pre-existing health inequities.

## Declarations of competing interest

The authors have no competing interest to declare.

## Appendix Supplementary materials

Image, application 1
