# Supplementary material for: Evaluation of risk prediction scores for adults hospitalized with COVID-19 in a highly-vaccinated population, Aotearoa New Zealand 2022
Source: IJID Reg. 2024 Aug 13;12:100424. doi: 10.1016/j.ijregi.2024.100424 (PMC11400985; doi:10.1016/j.ijregi.2024.100424)
Supplement: Supplementary file 1 [file mmc1.docx]

**Supplementary Material: Evaluation of risk prediction scores for adults hospitalised with COVID-19 in a highly vaccinated population, Aotearoa New Zealand 2022**

**Supplementary Methods. Attribution of hospital admission to COVID-19, Aotearoa New Zealand, 2022**

**Admission attribution to COVID-19:** During manual case review by a study clinician attribution of each hospital admission to COVID-19 was based on symptoms and diagnoses made by the treating medical team, according to the following definitions:

*Attributable to COVID-19-related illness or treatment of COVID-19:* Patients admitted due to symptoms caused primarily by SARS-CoV-2 infection. This includes: common viral symptoms, respiratory insufficiency, blood clot to vital organs, hemodynamic changes,^1^ as well as patients admitted due to an exacerbation of any underlying condition, where the treating clinician or reviewing physician consider COVID-19 contributory. This includes deterioration of: renal disease, cardiac disease, diabetes, dementia, and obstetric complications including pre-term birth and preeclampsia.

*Admission unrelated to COVID-19*: Admission history was unlikely to be related to COVID-19 and clinicians did not specifically admit the patient for COVID-19-related care. This admission could be due to, but not limited to: a) trauma, b) procedure or operation requiring hospitalisation, c) term labour, and d) alternative causes, including drug overdose, cancer progression, non-respiratory severe infection.^31^

*Admission unrelated to COVID-19, but subsequently contributed to hospital stay:* Admission was for an unrelated cause above, but subsequently met criteria of ‘Attributable to COVID-19-related illness or treatment of COVID-19.

**REFERENCE:**

1. Klann JG, Strasser ZH, Hutch MR, et al. Distinguishing admissions specifically for COVID-19 from incidental SARS-CoV-2 admissions: a National EHR research consortium study. J Med Internet Res 2022; 24(5): e37931.

**Supplementary Table S1. Demographic and admission characteristics of adults admitted to hospital due to COVID-19 by Māori, Pacific, and non-Māori non-Pacific, Aotearoa New Zealand, 2022**

| **Characteristic** | | **Māori (N=582)** | | **Pacific (N=914)** | **Non-Māori non-Pacific (N=862)** | | **Overall (N=2319)** |
| --- | --- | --- | --- | --- | --- | --- | --- |
|  | | **n (%)** | | **n (%)** | **n (%)** | | **n (%)** |
| Age (years), median (Q1, Q3) | | 52 (34, 65) | | 57 (37, 72) | 63 (40, 78) | | 57 (37, 74) |
| Female | | 358 (61·5%) | | 523 (57·2%) | 516 (59·9%) | | 1371 (59·1%) |
| Male | | 224 (38·5%) | | 391 (42·8%) | 346 (40·1%) | | 948 (40·9%) |
| Duration of symptoms on admission (days), median (Q1, Q3) | | 3·0 (1·0, 6·0) | | 3·0 (1·0, 6·0) | 3·0 (1·0, 6·0) | | 3·0 (1·0, 6·0) |
| Pregnant | | 50 (8·6%) | | 87 (9·5%) | 65 (7·5%) | | 198 (8·5%) |
| Number of COVID-19 vaccinations | |  | |  |  | |  |
|  | None | 131 (22·5%) | | 177 (19·4%) | 113 (13·1%) | | 415 (17.9%) |
|  | 1 | 24 (4·1%) | | 36 (3·9%) | 18 (2·1%) | | 76 (3.3%) |
|  | 2 | 230 (39·5%) | | 415 (45·4%) | 268 (31·1%) | | 896 (38.6%) |
|  | ≥3 | 197 (33·8%) | | 286 (31·2%) | 463 (53·7%) | | 932 (40.2%) |
| Smoking status | |  |  | |  | |  |
| Never smoked | | 148 (25·5%) | | 383 (42·0%) | | 467 (54·3%) | 988 (42·7%) |
| Ex-smoker | | 219 (37·7%) | | 288 (31·6%) | | 219 (25·5%) | 712 (30·8%) |
| Current smoker | | 163 (28·1%) | | 94 (10·3%) | | 68 (7·9%) | 314 (13·6%) |
| Unknown | | 51 (8·8%) | | 147 (16·1%) | | 106 (12·3%) | 301 (13·0%) |
| Immunosuppressive medications within last 3 months | | 51 (8·9%) | | 107 (11·8%) | 98 (11·4%) | | 252 (11·0%) |

**Supplementary Table S2. Calibration statistics of COVID-19 risk prediction scores among adults admitted to hospital due to COVID-19 by Māori, Pacific, and non-Māori non-Pacific, Aotearoa New Zealand, 2022**

| **Model** | **Measure** | **Original score** | **Recalibrated model (intercept and slope)** | **Re-estimated coefficients** |
| --- | --- | --- | --- | --- |
| **4C mortality** | Intercept | -1·45 (-1·63, -1·27) | -0·00 (-0·19, 0·19) | 0·08 (-0·11, 0·27) |
|  | Slope | 1·34 (1·16, 1·53) | 1·00 (0·86, 1·14) | 1·13 (0·98, 1·28) |
|  | C-index | 0·87 (0·84, 0·89) | 0·87 (0·84, 0·90) | 0·91 (0·88, 0·93) |
|  | Brier | 0·070 | 0·048 | 0·043 |
|  | Brier max | 0·059 | 0·059 | 0·059 |
|  | Brier scaled | -0·181 | 0·188 | 0·265 |
| **Modified PRIEST** | Intercept | -1·63 (-1·81, -1·45) | -0·00 (-0·18, 0·18) | 0·01 (-0·18, 0·19) |
|  | Slope | 0·85 (0·73, 0·99) | 1·00 (0·85, 1·15) | 1·01 (0·87, 1·15) |
|  | C-index | 0·83 (0·80, 0·85) | 0·83 (0·80, 0·85) | 0·87 (0·85, 0·90) |
|  | Brier | 0·086 | 0·053 | 0·049 |
|  | Brier max | 0·059 | 0·059 | 0·059 |
|  | Brier scaled | -0·458 | 0·097 | 0·164 |
| **CURB-65** | Intercept | 0·38 (0·20, 0·55) | -0·00 (-0·18, 0·18) | 0·10 (-0·09, 0·28) |
|  | Slope | 1·44 (1·23, 1·65) | 1·00 (0·85, 1·15) | 1·13 (0·99, 1·28) |
|  | C-index | 0·86 (0·82, 0·89) | 0·86 (0·82, 0·89) | 0·89 (0·86, 0·92) |
|  | Brier | 0·052 | 0·050 | 0·048 |
|  | Brier max | 0·059 | 0·059 | 0·059 |
|  | Brier scaled | 0·120 | 0·153 | 0·193 |
| **VACO (risk decile)** | Intercept | -0·63 (-0·81, -0·46) | 0·00 (-0·17, 0·17) | -0·00 (-0·18, 0·18) |
|  | Slope | 0·67 (0·53, 0·82) | 1·00 (0·78, 1·22) | 1·00 (0·82, 1·18) |
|  | C-index | 0·79 (0·75, 0·83) | 0·79 (0·75, 0·83) | 0·83 (0·78, 0·86) |
|  | Brier | 0·060 | 0·055 | 0·053 |
|  | Brier max | 0·059 | 0·059 | 0·059 |
|  | Brier scaled | -0·012 | 0·069 | 0·097 |

**Supplementary Table S3. Odds ratios for predictors of mortality in the 4C mortality score in the original and re-estimated models**

|  | | **Original** |  | **Re-estimated model** | | |
| --- | --- | --- | --- | --- | --- | --- |
| **Predictor** | **Level** | **Odds ratio** |  | **Odds ratio** | **95% CI** | **p** |
| Model intercept (baseline risk) | - | 0·01 |  | 0 | 0·00, 0·00 | <0·001 |
| Age (years) | 50-59 | 1·99 |  | 2·38 | 0·94, 6·02 | 0·068 |
|  | 60-69 | 3·81 |  | 2·57 | 1·06, 6·21 | 0·036 |
|  | 70-79 | 6·31 |  | 2·57 | 1·08, 6·14 | 0·033 |
|  | >80 | 9·51 |  | 6·77 | 3·00, 15·31 | <0·001 |
| Sex | Male | 1·19 |  | 1·27 | 0·85, 1·91 | 0·238 |
| Number of comorbidities | 1 | 1·35 |  | 4·27 | 1·51, 12·04 | 0·006 |
|  | ≥2 | 1·7 |  | 4·27 | 1·57, 11·62 | 0·005 |
| Respiratory rate (breaths/min) | 20-29 | 1·26 |  | 1·68 | 1·02, 2·77 | 0·042 |
|  | ≥30 | 1·91 |  | 1·68 | 0·84, 3·38 | 0·145 |
| Oxygen saturation < 92% | - | 1·78 |  | 2·05 | 1·15, 3·63 | 0·015 |
| Glasgow coma scale < 15 | - | 1·75 |  | 6·71 | 4·09, 11·02 | <0·001 |
| Urea (mmol/L) | 7-14 | 1·55 |  | 1·91 | 1·14, 3·20 | 0·013 |
| Urea (mmol/L) | >14 | 2·75 |  | 2·74 | 1·55, 4·84 | <0·001 |
| C-reactive protein (mg/L) | 50-99 | 1·44 |  | 2·79 | 1·66, 4·70 | <0·001 |
| C-reactive protein (mg/L) | ≥100 | 2·1 |  | 2·83 | 1·68, 4·78 | <0·001 |

**Supplementary Table S4. Odds ratios for predictors of mortality in the CURB-65 score in re-estimated models**

|  |  | **Original** |  | **Re-estimated model** | | |
| --- | --- | --- | --- | --- | --- | --- |
| **Predictor** | **Level** | **Odds ratio** |  | **Odds ratio** | **95% CI** | **p** |
| Model intercept (baseline risk) | - | n/a |  | 0·01 | 0·01, 0·01 | <0·001 |
| Age >65 years | n/a | n/a |  | 2·37 | 1·51, 3·73 | <0·001 |
| Blood pressure | SBP <90 mmHg or DBP ≤60 mmHg | n/a |  | 2·85 | 1·82, 4·45 | <0·001 |
| Urea | >7·0 mmol/L | n/a |  | 3·22 | 2·07, 4·99 | <0·001 |
| GCS<15 | <15 | n/a |  | 7·46 | 4·68, 11·88 | <0·001 |
| Respiratory rate | ≥30 | n/a |  | 1·51 | 0·87, 2·60 | 0·139 |

Abbreviations: SBP= systolic blood pressure; DBP= diastolic blood pressure

**Supplementary Table S5. Odds ratios for predictors of mortality in the modified PRIEST score in the original and re-estimated models**

|  | | **Original** |  | **Re-estimated model** | | |
| --- | --- | --- | --- | --- | --- | --- |
| **Predictor** | **Level** | **Odds ratio** |  | **Odds ratio** | **95% CI** | **p** |
| Model intercept (baseline risk) | - | 0·02 |  | 0 | 0·00, 0·01 | <0·001 |
| Sex | Male | 1·38 |  | 1·42 | 0·97, 2·07 | 0·073 |
| Age (years) | 50-65 | 2·08 |  | 3·82 | 1·68, 8·70 | 0·001 |
|  | 66-80 | 2·25 |  | 5·28 | 2·37, 11·79 | <0·001 |
|  | >80 | 2·56 |  | 16·38 | 7·45, 36·04 | <0·001 |
| Respiratory rate (breaths/min) | 21-24 | 1·4 |  | 1·23 | 0·76, 1·98 | 0·403 |
|  | <9 or >24 | 2·08 |  | 1·23 | 0·73, 2·07 | 0·445 |
| Oxygen saturation | 94-95% | 1·43 |  | 1·13 | 0·64, 2·00 | 0·663 |
|  | 92-93% | 1·97 |  | 1·17 | 0·57, 2·39 | 0·673 |
|  | <92% | 3·16 |  | 1·28 | 0·67, 2·46 | 0·453 |
| Heart rate, beats per min | 41-50 or 91-110 | 0·94 |  | 1·16 | 0·75, 1·80 | 0·494 |
|  | 111-130 | 1·14 |  | 1·16 | 0·58, 2·33 | 0·668 |
|  | <41 or >130 | 1·4 |  | 1·45 | 0·56, 3·79 | 0·444 |
| Systolic BP, mmHg | 91-100 | 1·85 |  | 1·67 | 0·91, 3·06 | 0·099 |
|  | 101-110 | 1·25 |  | 2·76 | 1·44, 5·25 | 0·002 |
|  | <91 or >219 | 1·86 |  | 3·22 | 1·53, 6·79 | 0·002 |
| Temperature | <35·1 | 2·4 |  | 1·44 | 0·39, 5·41 | 0·584 |
|  | 35·1-36·0 or 38·1-39·0 | 1·3 |  | 1·00 | 0·66, 1·53 | >0·999 |
|  | >39·0 | 1·15 |  | 1·00 | 0·38, 2·61 | >0·999 |
| Alertness | Confused or not alert | 1·49 |  | 2·13 | 1·39, 3·24 | <0·001 |
| Supplemental oxygen | - | 3·43 |  | 4·97 | 3·07, 8·04 | <0·001 |

Abbreviations: BP = blood pressure

**Supplementary Table S6. Odds ratios for predictors of mortality in the VACO index in the original and re-estimated models**

|  | | **Original** |  | **Re-estimated model** | | |
| --- | --- | --- | --- | --- | --- | --- |
| **Predictor** | **Level** | **Odds ratio** |  | **Odds ratio** | **95% CI** | **p** |
| Model intercept (baseline risk) | - | 0·01 |  | 0 | 0·00, 0·02 | <0·001 |
| Sex | Male | 1·38 |  | 1·5 | 1·05, 2·17 | 0·028 |
| Age (years) | [16,50) | 0·11 |  | 0·72 | 0·22, 2·40 | 0·595 |
|  | [55,60) | 1·49 |  | 1·00 | 0·31, 3·18 | >0·999 |
|  | [60,65) | 2·56 |  | 1·00 | 0·33, 3·03 | >0·999 |
|  | [65,70) | 3·65 |  | 1·00 | 0·34, 2·93 | >0·999 |
|  | [70,75) | 5·1 |  | 1·00 | 0·34, 2·92 | >0·999 |
|  | [75,80) | 5·83 |  | 1·00 | 0·35, 2·88 | >0·999 |
|  | [80,90) | 6·87 |  | 1·75 | 0·63, 4·85 | 0·285 |
|  | [90,100] | 7·53 |  | 1·75 | 0·51, 5·97 | 0·374 |
| History of MI or PVD | - | 1·31 |  | 1·00 | 0·63, 1·59 | >0·999 |
| Age and CCI* | Age <85 & CCI 1-3 | 1·84 |  | 4·29 | 0·82, 22·5 | 0·086 |
|  | Age <85 & CCI 4-5 | 2·28 |  | 12·67 | 2·38, 67·5 | 0·003 |
|  | Age <85 & CCI 6-9 | 2·6 |  | 32·34 | 6·11, 171 | <0·001 |
|  | Age <85 & CCI 10+ | 4·04 |  | 32·83 | 5·08, 212 | <0·001 |
|  | Age 85+ & CCI Any | 4·62 |  | 34·42 | 5·88, 201 | <0·001 |

Key: * Interaction term; Abbreviations: CCI = Charlson comorbidity index, MI = myocardial infarction, PVD = peripheral vascular disease

**Supplementary Table S7. Estimated concordance-statistic (95% confidence intervals) using complete case analysis of selected severity prediction scores in adults admitted to hospital due to COVID-19 by Māori, Pacific, and non-Māori non-Pacific, Aotearoa New Zealand, 2022**

| **Model** | **All** | **Māori** | **Pacific** | **Non-Māori, non-Pacific** |
| --- | --- | --- | --- | --- |
| 4C mortality | 0.88 (0.82, 0.92) | 0.86 (0.71, 0.94) | 0.87 (0.73, 0.95) | 0.89 (0.79, 0.95) |
| Modified PRIEST | 0.81 (0.77, 0.85) | 0.86 (0.80, 0.91) | 0.78 (0.70, 0.85) | 0.81 (0.75, 0.86) |
| CURB65 | 0.86 (0.76, 0.93) | 0.88 (0.35, 0.99) | 0.85 (0.70, 0.93) | 0.86 (0.67, 0.95) |
| VACO | 0.79 (0.75, 0.83) | 0.72 (0.58, 0.82) | 0.78 (0.72, 0.83) | 0.83 (0.77, 0.87) |

**Supplementary Table S8. Performance metrics of the 4C mortality score in patients aged ≥16 years admitted to hospital due to COVID-19, New Zealand, 2022**

| **Threshold** | **n (%)** | **TP** | **TN** | **FP** | **FN** | **Sensitivity (%)** | **Specificity (%)** | **PPV (%)** | **NPV (%)** | **Mortality (%)** |
| --- | --- | --- | --- | --- | --- | --- | --- | --- | --- | --- |
| <2 | 328 (14·1) | 146 | 328 | 1845 | 0 | 100 | 15·1 | 7·3 | 100 | 0 |
| <4 | 735 (31·7) | 146 | 735 | 1438 | 0 | 100 | 33·8 | 9·2 | 100 | 0 |
| <6 | 997 (43·0) | 143 | 994 | 1179 | 3 | 97·9 | 45·7 | 10·8 | 99·7 | 0·3 |
| <8 | 1282 (55·3) | 135 | 1271 | 902 | 11 | 92·5 | 58·5 | 13·0 | 99·1 | 0·9 |
| <10 | 1594 (68·7) | 123 | 1571 | 602 | 23 | 84·2 | 72·3 | 17·0 | 98·6 | 1·4 |
| <12 | 1921 (82·8) | 96 | 1871 | 302 | 50 | 65·8 | 86·1 | 24·1 | 97·4 | 2·6 |
| <14 | 2141 (92·3) | 64 | 2059 | 114 | 82 | 43·8 | 94·8 | 36 | 96·2 | 3·8 |
| <16 | 2258 (97·4) | 32 | 2144 | 29 | 114 | 21·9 | 98·7 | 52·5 | 95·0 | 5·0 |
| <18 | 2302 (99·3) | 12 | 2168 | 5 | 134 | 8·2 | 99·8 | 70·6 | 94·2 | 5·8 |
| <20 | 2314 (99·8) | 3 | 2171 | 2 | 143 | 2·1 | 99·9 | 60 | 93·8 | 6·2 |

Key: n= number of patients, TP= true positive, TN = true negative, FP= false positive, FN= false negative, PPV= positive predictive value, NPV= negative predictive value

**Supplementary Table S9. Performance metrics of the modified PRIEST score in patients aged ≥16 years admitted to hospital due to COVID-19, New Zealand, 2022**

| **Threshold** | **n (%)** | **TP** | **TN** | **FP** | **FN** | **Sensitivity (%)** | **Specificity (%)** | **PPV (%)** | **NPV (%)** | **Mortality (%)** |
| --- | --- | --- | --- | --- | --- | --- | --- | --- | --- | --- |
| <2 | 275 (11·9) | 146 | 275 | 1898 | 0 | 100 | 12·7 | 7·1 | 100 | 0 |
| <4 | 710 (30·6) | 146 | 710 | 1463 | 0 | 100 | 32·7 | 9·1 | 100 | 0 |
| <6 | 1199 (51·7) | 137 | 1190 | 983 | 9 | 93·8 | 54·8 | 12·2 | 99·2 | 0·8 |
| <8 | 1597 (68·9) | 118 | 1569 | 604 | 28 | 80·8 | 72·2 | 16·3 | 98·2 | 1·8 |
| <10 | 1859 (80·2) | 92 | 1805 | 368 | 54 | 63·0 | 83·1 | 20·0 | 97·1 | 2·9 |
| <12 | 2087 (90·0) | 57 | 1998 | 175 | 89 | 39·0 | 91·9 | 24·6 | 95·7 | 4·3 |
| <14 | 2215 (95·5) | 31 | 2100 | 73 | 115 | 21·2 | 96·6 | 29·8 | 94·8 | 5·2 |
| <16 | 2273 (98·0) | 18 | 2145 | 28 | 128 | 12·3 | 98·7 | 39·1 | 94·4 | 5·6 |
| <18 | 2301 (99·2) | 11 | 2166 | 7 | 135 | 7·5 | 99·7 | 61·1 | 94·1 | 5·9 |
| <20 | 2315 (99·8) | 3 | 2172 | 1 | 143 | 2·1 | 100 | 75 | 93·8 | 6·2 |

Key: n= number of patients, TP= true positive, TN = true negative, FP= false positive, FN= false negative, PPV= positive predictive value, NPV= negative predictive value

**Supplementary Table S10. Performance metrics of the CURB-65 score in patients aged ≥16 years admitted to hospital due to COVID-19, New Zealand, 2022**

| **Threshold** | **n (%)** | **TP** | **TN** | **FP** | **FN** | **Sensitivity (%)** | **Specificity (%)** | **PPV (%)** | **NPV (%)** | **Mortality (%)** |
| --- | --- | --- | --- | --- | --- | --- | --- | --- | --- | --- |
| 0 | 910 (39.2) | 145 | 909 | 1264 | 1 | 99.3 | 41.8 | 10.3 | 99.9 | 0.1 |
| ≤1 | 1573 (67.8) | 125 | 1552 | 621 | 21 | 85.6 | 71.4 | 16.8 | 98.7 | 1.3 |
| ≤2 | 2048 (88.3) | 75 | 1977 | 196 | 71 | 51.4 | 91 | 27.7 | 96.5 | 3.5 |
| ≤3 | 2259 (97.4) | 30 | 2143 | 30 | 116 | 20.5 | 98.6 | 50 | 94.9 | 5.1 |
| ≤4 | 2315 (99.8) | 3 | 2172 | 1 | 143 | 2.1 | 100 | 75 | 93.8 | 6.2 |

Key: n= number of patients, TP= true positive, TN = true negative, FP= false positive, FN= false negative, PPV= positive predictive value, NPV= negative predictive value
